# An exploratory pilot study to assess self-perceived changes among social assistance recipients regarding employment prospects after receiving dental treatment

**DOI:** 10.1186/s12903-015-0119-2

**Published:** 2015-11-04

**Authors:** Sonica Singhal, Muhammad Mamdani, Andrew Mitchell, Howard Tenenbaum, Carlos Quiñonez

**Affiliations:** Discipline of Dental Public Health, Faculty of Dentistry, University of Toronto, Toronto, ON Canada; Li Ka Shing Knowledge Institute, St. Michael’s Hospital, University of Toronto, Toronto, ON Canada; Faculty of Social Work, University of Toronto, Toronto, ON Canada; Hospital for Sick Children, Department of Pediatrics, Faculty of Medicine, University of Toronto, Toronto, ON Canada

**Keywords:** Unemployment, Oral health, Self-efficacy, Welfare, Dental treatment

## Abstract

**Background:**

Strengthening self-efficacy in job-seeking among individuals with dental problems has been identified as an important factor in facilitating job procurement and maintenance. There is no knowledge about whether receiving dental treatment improves someone’s self-efficacy in seeking a job. This work explores this relationship.

**Methods:**

An exploratory pilot study of a convenience sample of 30 social assistance recipients of Ontario, Canada, was conducted using a pre- and post-dental treatment survey, which included both quantitative and qualitative components. The survey included two validated instruments Oral Health Impact Profile (OHIP-14) and Job-Seeking Self-efficacy scale (JSS). Changes in scores of both scales following dental treatment were calculated. Pearson correlation was performed between OHIP-14 and JSS scores. Qualitative data were transcribed and interrelated ideas were grouped together to generate themes.

**Results:**

Mean scores for OHIP-14 (23.4 to 6.7, *p* < 0.001, effect size: 1.75) and median scores for JSS (4.9 to 5.5, *p* = 0.002, effect size: 0.40) changed significantly after receiving dental treatment. A significant negative correlation (−0.56, *p* = 0.001) was observed between OHIP-14 and JSS scores indicating that job-seeking self-efficacy improves with improvement in oral health related quality of life (OHRQoL). Qualitative analysis reveals participants’ physical and psychosocial impacts of dental problems; barriers experienced in accessing dental care and seeking a job; and changes perceived after receiving dental care.

**Conclusion:**

Results of our survey indicate that social assistance recipients experience negative impacts of dental problems and perceive improvements in OHRQoL and job-seeking self-efficacy after receiving dental treatment.

**Electronic supplementary material:**

The online version of this article (doi:10.1186/s12903-015-0119-2) contains supplementary material, which is available to authorized users.

## Background

According to Locker’s model of oral health, dental diseases lead to physical, psychological and social disability, influencing the way people eat, speak and/or socialize [1]. Among people who experience some level of social marginalization, the association of psychosocial function and dental health appears to be particularly strong [2, 3]. In this context, the association of such disabilities to an individual’s employment situation has also received attention [4–6].

Oral diseases are distributed disproportionately in society and are concentrated among populations with low socioeconomic status. One such disadvantaged group includes people on social assistance, who along with poor oral health, also experience economic consequences as a result of oral disease [7, 8]. In this regard, policy entrepreneurs in welfare states have advocated for improved access to dental care for people on social assistance not only from an ethical perspective, but also to improve their prospects of employment [2]. Since little to no evidence exists to support this policy hypothesis, we conducted a population-based study among social assistance recipients in Ontario, Canada’s most populated province, to compare employment outcomes among those who received and did not receive dental treatment under the province’s social assistance program, Ontario Works (OW). Our study suggested that people who received dental treatment were more disadvantaged at baseline, and that treatment may have addressed their employment barriers to some extent, such that employment outcomes were levelled up over time [9]. This leads us to question what employment barriers may be addressed by receiving dental treatment? What are the self-perceived changes in terms of employment after receiving treatment for dental problems among this population? Does dental treatment, for example, affect self-efficacy among those seeking a job?

People with disabilities (including dental disabilities) who are unemployed may lack the necessary job-seeking skills required to secure employment. Strengthening self-efficacy in job-seeking among individuals with disabilities has been identified as an important factor in facilitating job procurement and maintenance [10]. Psychologist Albert Bandura defined self-efficacy as one’s belief in one’s capacity to mobilize the physical, emotional, and intellectual resources required to succeed in specific situations [11]. Feeling efficacious motivates intensification of efforts and persistence. The stronger the perceived self-efficacy, the more active the efforts are, whereas low levels of self-efficacy tends to result in self-limiting behaviors that create obstacles to new experiences.

The relationship between employment and self-efficacy has been demonstrated in the literature [12, 13]. For example, self-efficacy and self-esteem are intimately involved with unemployment. Self-esteem declines with unemployment and, in turn, affects self-efficacy. Securing employment acts as a restorative measure and enables self-efficacy to rebound.

**Fig. 1:**
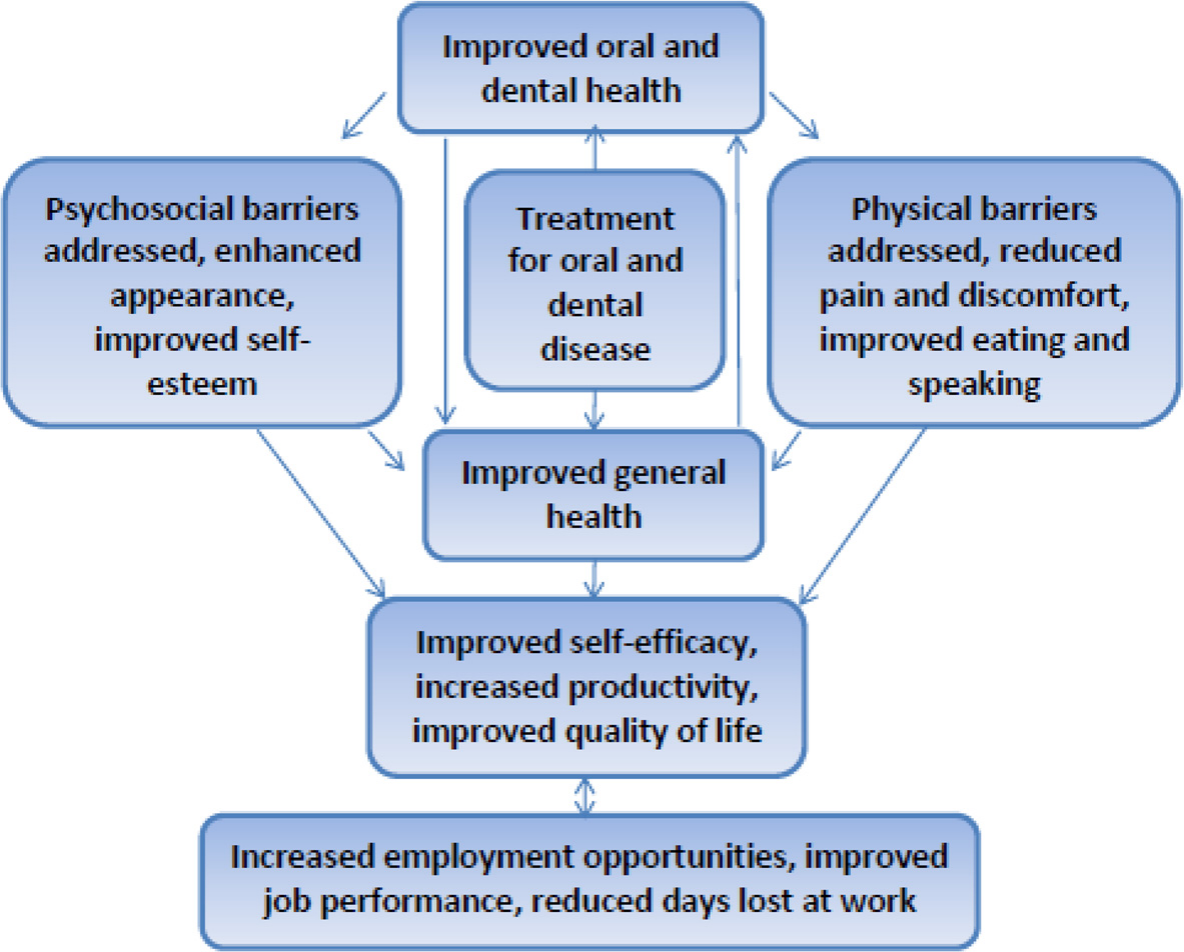
Conceptual pathways of receiving dental care to employment and quality of life outcomes

Again, there is very little knowledge regarding how receiving dental treatment may mediate positive employment outcomes for disadvantaged populations. Based on the existing literature, we designed a conceptual framework that depicts the association of dental care and employment outcomes (Fig. 1). According to this framework, addressing physical, pathological and psychological impacts of dental problems has the potential to improve oral health related quality of life (OHRQoL) and the efficacy of job-seeking, which could lead to positive employment outcomes. Our study was an endeavour to explore the effect of dental treatment on OHRQoL and job-seeking self-efficacy among patients on social assistance.

## Methods

We hypothesized that for people on social assistance who have dental problems; the provision of dental treatment would improve OHRQoL and thereby improve their self-efficacy of job-seeking. Specific objectives were to observe changes in OHRQoL and job-seeking self-efficacy after receiving dental treatment; and to determine whether there is a correlation between changes in two aspects.

### Study design

A prospective non-randomized clinical study was designed to conduct a pre- and post-dental treatment survey among people on social assistance. A fifty-item questionnaire was designed in consultation with officials at Toronto Employment and Social Services; this questionnaire was administered to assess OHRQoL, self-perceived dental needs, and job-seeking self-efficacy. Information was also collected on: age; sex; general health conditions; habits such as smoking, alcohol, recreational drugs, gambling; family structure; type of accommodation; number of children in care and their age; born in or outside Canada, and if outside, for how long have they been in the country. Qualitative data were also collected through open-ended questions to have a better understanding regarding issues related to participants’ OHRQoL and employment.

### Instruments used

1. *Oral Health Impact Profile (OHIP-14)* – This is a validated fourteen item instrument developed to measure people’s perception of the impact of oral diseases on their well-being [14, 15]. It assesses the OHRQoL of a patient and has been shown to be sensitive (i.e. significant changes in scores) to the effects of the provision of dental care [16]. According to Locker et al., a change of five points on the scale is a minimally important difference [16]. The OHIP-14 has been shown to be reliable, sensitive to changes, and has adequate cross-cultural consistency.
  The frequency of the impact experienced is recorded on a five-point Likert scale: 0 = ‘never’; 1 = ‘hardly ever’; 2 = ‘occasionally’; 3 = ‘fairly often’; and 4 = ‘very often’ (Additional file 1). For analysis, these ordinal values can be computed three ways: prevalence - proportion of participants reporting one or more items ‘fairly often’ or ‘very often’; extent - number of items out of fourteen reported ‘fairly often’ or ‘very often’; and severity - sum of ordinal responses, which additionally considers impacts experienced ‘hardly ever’ or ‘occasionally’ and could range from 0 to 56 [17]. To take into consideration the complete account of OHIP-14 scores, severity was computed.
2. *Job-seeking Skills Self-efficacy (JSS) scale* - The JSS is a validated twelve item scale to assess the perceived influence of self-efficacy on the job-seeking skills of persons with chronic disabilities and has been previously used for people with arthritis [18] (Additional file 2). Dental disease is chronic in nature and leads to physical, psychological and social disability [1]; therefore, the JSS scale was considered a useful tool to assess job-seeking self-efficacy among people with dental disabilities. The instrument was included in pre- and post-surveys to assess changes in job-seeking self-efficacy after receiving dental treatment. The scale has two factors, independence skills (IS) and social skills (SS). The IS factor is a summated scale of the first five items and the SS factor is a summated scale of the rest of the seven items of the JSS. All questions are assessed using a seven-point Likert scale of where 1 is ‘not at all confident’ and 7 is ‘very confident’.

### Study participants

Clients of OW (age range 18–65 years), who had any kind of dental problem and who sought dental care at public health clinics in the City of Toronto, at dental clinics of Faculty of Dentistry, University of Toronto, or at Community Health Centers (CHCs) in Toronto were selected for this study. Ethics approval for conducting this research was obtained from the Research Ethics Board of the University of Toronto (protocol reference # 30174) and also from Toronto Public Health (file # 2014–17). An invitation letter for the participants, describing the purpose of the study and contact information of the principal investigator (PI) was made available at reception desks and notice boards. Potential subjects who phoned the PI were provided further details and screened through simple inclusion criteria questions (social assistance status, age, and having a dental problem). They were then invited to the Faculty of Dentistry for a face-to-face survey. The interviews were conducted in an isolated, well lit room, with comfortable chairs, where only the PI and the interviewee were present. Participation was strictly voluntary. A thorough verbal explanation of the study, along with a hard copy of its details was provided to all participants. Those who wished to participate were then enrolled and a written informed consent was signed. A second interview was conducted after one month of receiving dental treatment, which has been reported as being a reasonable time gap to conduct a post-treatment survey [16]. The same item questionnaire was re-administered, asking if any demographic or behavioral factors had changed.

We know of no study that has tried to assess employment-related psychosocial changes after receiving dental treatment. Therefore, ours was a pilot study, having both a quantitative and a qualitative component, with a convenience sample size of 30 participants. As per Hardon et al., for descriptive studies, a sample size of at least a sample of 30 people is needed; it needs to be large enough to reflect important variations in the study population, but small enough to facilitate intensive study [19]. To increase the recruitment and retention of participants, we provided an honorarium of CAD 50 for their time at each appointment.

### Statistical analysis

Descriptive statistics for all demographic variables was conducted by univariate analysis. Shapiro-Wilk test of normality was conducted for the distribution of data of the JSS and OHIP-14 scores. If data were normally distributed, the paired *t*-test was used; otherwise, the Wilcoxon signed rank sum test was conducted. Effect sizes for both scales to assess changes after receiving dental treatment were calculated. For normally distributed data, mean change scores were calculated by subtracting the baseline scores from those at follow-up; effect size was calculated by dividing the mean change scores by baseline standard deviation. For data that were not normally distributed, the standard score (z) was divided by the square root of N (*N* = number of observations), to calculate the effect size. According to Cohen, an effect size of 0.2 is considered small, 0.5 as moderate, and 0.8 and above as large [20]. Pearson product correlation was also performed between JSS and OHIP scores to assess if change in OHRQoL e was correlated to job-seeking self-efficacy. Pearson correlation ranges from −1 to +1, where the sign indicates the direction of correlation and the value indicates the magnitude; 0.5–0.7 is considered moderate and 0.7–1.0 is considered strong [21].

Qualitative data were collected utilizing standard interview techniques, specifically a dialogical interview process [22]. These data were hand transcribed, and then major themes were identified. Member checking was conducted by reading the responses back to the interviewee to make sure that responses were not misreported. Interrelated ideas were grouped together to generate themes. Themes are fundamental concepts that characterize experiences of participants by the more general insights that are apparent from the whole of the data [23].

## Results

Participants were enrolled over a time period of eight months (April 2014 – November 2014). Thirty-four OW clients were invited by the PI to participate in the study. In terms of baseline demographic information (Table 1), the ratio of men to women was approximately 40:60; mean age of participants was 48 years; 35 % were single with children and 20 % had children less than four years of age.

**Table 1:** Baseline demographic characteristics of study participants

Demographics	Proportions (%)
	(Total *N* = 34)
Male	41.20 %
Age	47.7 (± SD: 11.3)
Born in Canada	38.20 %
Has child < = 4 years	20.60 %
On social assistance >4 years	32.40 %
Educational level < than high school	8.80 %
Single with children	35.30 %
Couple with children	17.60 %
No children	47.10 %

Self-perceived dental needs indicate that, at baseline, a large proportion (85 %) of participants accessed dental care in the case of emergency (Table 2). Most visited a dentist because of severe pain, with some also experiencing swelling along with that pain. Extraction or root canal treatment was advised to most who sought emergency dental care. Sixty-five percent of participants felt the need for dentures (combining removable and fixed). At follow-up, out of the 34 participants: 22 felt they had all their dental needs met post-treatment; eight had their partial dental needs met; and for three, no dental needs were met. One client was lost to follow up. Therefore 30 clients, who had their full or partial dental needs met, were interviewed for a second time. Among the eight clients, whose dental needs were partially met, six could not receive their dentures and two could not get their teeth cleaned. Among the three clients, whose dental needs were not met at all, all needed dentures and had continuing problems with their front teeth. Financial barriers were the only reason identified by all participants, whose dental needs were not met (completely or partially).

**Table 2:** Self-perceived dental needs of study participants at baseline (*N* = 34)

Type of treatment	Proportion
Emergency	85 %
Fillings	44 %
Gum problem	12 %
Extractions	53 %
Root canal treatment	47 %
Dentures	65 %
Preventive	41 %
Treatment of front teeth	38 %

*The proportions do not add up to 100 % as participants had more than one type of treatment needs

According to the Shapiro Wilk test of normality, change in OHIP-14 scores was normally distributed (*p* = 0.25); however, JSS scores were not (*p* < 0.001). Mean OHIP-14 scores reduced from 23.4 (SD = 11.83) to 6.7 (SD = 6.45), reflecting that participants perceived better OHRQoL after receiving dental treatment. This change was statistically significant (*p* < 0.001) and the effect size was 1.75.

Total median JSS scores also increased significantly after receiving dental treatment suggesting that the study participant’s job-seeking self-efficacy improved (Table 3). These improvements were consistent for both independence skills and social skills. Moderate effect sizes for total JSS as well as for each individual construct were observed.

**Table 3:** Median values of job seeking self-efficacy skills (pre- and post-dental treatment)

Job seeking self-efficacy scale (JSS)	Median values (interquartile range)		Effect size (*p* value)
	Pre-treatment	Post-treatment	
Total scale	5.0 (4.2–6.0)	5.5 (4.8–6.5)	D = 0.40 (*p* = 0.002)
Independence skills	5.4 (4.8–6.6)	5.8 (5.0–6.7)	D = 0.34 (*p* = 0.011)
Social skills	4.9 (3.2–6.0)	5.4 (4.7–6.3)	D = 0.39 (*p* = 0.003)

Pearson correlational analysis revealed a significant negative association between change in OHIP scores and change in JSS scores, which means that, if dental disability among an individual reduces, job-seeking self-efficacy improves (Table 4). For JSS, when stratified by social and independence skills, only social skills were significantly associated with OHIP score changes.

**Table 4:** Pearson correlation between changes in OHIP and JSS scores

Change in JSS	Correlation with change in OHIP-14 (*p* value)
Total JSS scores	−0.56 (0.001)
Independence skills	−0.15 (0.418)
Social skills	−0.62 (<0.001)

Qualitative data helped us to understand different aspects related to participants OHRQoL and employment prospects. In addition to responses to survey questions, some participants provided written narratives of their experiences. The four main themes, which emerged, are as follows:

### Physical and psychosocial impacts of dental problems

Participants perceived their poor OHRQoL as a major barrier to their social life. Embarrassment associated and the distressing impact on self-esteem was discussed. Such experiences are in accordance with the a study done in Canada by Bedos et al. [2].

### Financial barriers experienced in accessing dental care

Most of the participants discussed the financial hardship they experienced in accessing dental care. Without enough money of their own, and with publicly funded dental services limited to emergency, the respondents shared their struggle with finding ways to receive cheaper services. Some participants shared their provider’s generous dental financial aid schemes such as monthly payment plan, which facilitated their timely treatment. One of the participants stopped going to his regular dentist, as the latter charged patients upfront and did not deal with any dental insurances directly.

### Changes encountered after receiving dental care

Some participants shared the changes they perceived after receiving dental treatment. In particular, participants who received dentures were very excited to share their stories.

### Other barriers encountered in seeking a job

Apart from poor OHRQoL, increasing age, low education, young children, lack of English language proficiency, and poor general health were the main barriers perceived by study participants in seeking a job. Among health issues, mental health and arthritis were the major concerns.

## Discussion

Face-to-face pre- and post-treatment interviews were conducted among social assistance recipients in Ontario to assess if dental treatment helped in improving OHRQoL and in turn enhancing job-seeking self-efficacy. The dental needs of study participants were assessed by self-report and no clinical examinations were performed. Data collection was primarily quantitative in nature and qualitative component was basic in methodological robustness; however, participants’ responses were very informative and corroborated findings of quantitative analysis. This was the first such work conducted in the Canadian context. Participants’ responses reflect the vulnerability of this social group, and suggest that dental treatment can address physical as well as psychosocial disabilities related to dental problems. Change in mean OHIP-14 scores is in accordance with other studies [24, 25]. Job-seeking self-efficacy also appears to have enhanced after receiving dental care.

The effect size for change in OHIP-14 score was large; however, for JSS score it was moderate. Interestingly, Pearson correlation revealed that change in social skills but not independence skills was significantly associated with change in OHIP-14 scores. These results are understandable, as it is arguably naïve to think that improvements in job-seeking self-efficacy could be completely attributable to improvements in OHRQoL. A substantial interplay of other individual and social determinants with OHRQoL and employment outcomes of our study participants was expected and was confirmed through the qualitative component of our study. Factors such as the inability to eat and speak, feelings of persistent self-consciousness and social exclusion, financial struggles to make choices between dental treatment and daily utilities, poor general health including mental health issues, and dependence on recreational drugs emerged, which were perceived by participants as barriers to their employment and also to improvements in their OHRQoL. This study suggests that timely dental care can, to a certain extent, address employment barriers. From the policy perspective, reducing an individual deficiency (which is poor OHRQoL in this context) to improve employment opportunities is only one aspect of handling social determinants of health; however, addressing poor OHRQoL cannot be discounted, especially when we now know that improving OHRQoL affects self-efficacy, which has long term positive impacts in general.

Our study though recruited participants from three different sites, it included a non-probabilistic sample of only thirty participants; therefore, results of this study cannot be generalized and should be extrapolated for other populations with caution. If larger sample size could be recruited, a control group would have been established to observe if OHRQoL and JSS changes over time without dental intervention. Also, it would have been interesting to observe if JSS deteriorates, for those, who could not seek required dental treatment. Nonetheless, the effect sizes calculated through this study will certainly be useful in conducting a larger study in the future. Ultimately, being face-to-face surveys, these results can be subjected to reporting bias as participants responses might be modified in response to their awareness of being observed. However, being a mixed method study, administering face-to-face surveys were considered most suitable to have a thorough understanding regarding different aspects.

## Conclusion

Our results suggest that after receiving dental treatment, OHRQoL and job-seeking self-efficacy may improve. Importantly, with the effect sizes calculated, larger population-based studies can now be planned to more robustly test the role of dental treatment on the employment outcomes of social assistance recipients. Results of such studies can inform policy and advocacy efforts at expanding public dental care programs for low-income adults, partly because of the role that dental treatment can play on improving employment outcomes.

## Additional files

Additional file 1:
**Table.** Oral Health Impact Profile -14 (OHIP-14) questionnaire. (DOCX 18 kb) Additional file 2:
**Table.** Job-seeking Skills Self-efficacy (JSS) scale. (DOCX 58 kb)

## Abbreviations

OW: Ontario Works
CHCs: Community Health Centers
PI: Principal investigator
OHRQoL: Oral Health Related Quality of Life
OHIP 14: Oral Health Impact Profile
JSS: Job-seeking Skills Self-efficacy
IS: Independence skills
SS: Social skills
