# Supplementary material for: An exploratory pilot study to assess self-perceived changes among social assistance recipients regarding employment prospects after receiving dental treatment
Source: BMC Oral Health. 2015 Nov 4;15:138. doi: 10.1186/s12903-015-0119-2 (PMC4632367; doi:10.1186/s12903-015-0119-2)
Supplement: Additional file 1: — Table. Oral Health Impact Profile -14 (OHIP-14) questionnaire. (DOCX 18 kb) [file 12903_2015_119_MOESM1_ESM.docx]

**Appendix1**

**Table: Oral Health Impact Profile -14 (OHIP-14) questionnaire**

| Dimension | Questions |
| --- | --- |
| Functional limitation | Have you had *trouble pronouncing any words*, because of problems with your teeth, mouth or dentures? |
|  | Have you felt that *your sense of taste has worsened* because of problems with your teeth, mouth or dentures? |
| Physical pain | Have you had *painful aching* in your mouth? |
|  | Have you found *uncomfortable to eat any foods* because of problems with your teeth, mouth or dentures? |
| Psychological discomfort | Have you been *self-conscious* because of your teeth, mouth or dentures? |
|  | Have you *felt tense* because of problems with your teeth, mouth or dentures? |
| Physical disability | Has your *diet been unsatisfactory* because of problems with your teeth, mouth or dentures? |
|  | Have you had to *interrupt meals* because of problems with your teeth, mouth or dentures? |
| Psychological disability | Have you find it *difficult to relax* because of problems with your teeth, mouth or dentures? |
|  | Have you been a *bit* *embarrassed* because of problems with your teeth, mouth or dentures? |
| Social disability | Have you been a *bit irritable with other people* because of problems with your teeth, mouth or dentures? |
|  | Have you had *difficulty doing your usual jobs* because of problems with your teeth, mouth or dentures? |
| Handicap | Have you felt that *life in general was less satisfying* because of problems with your teeth, mouth or dentures? |
|  | Have you been *totally unable to function* because of problems with your teeth, mouth or dentures? |
