# Supplementary material for: An exploratory pilot study to assess self-perceived changes among social assistance recipients regarding employment prospects after receiving dental treatment
Source: BMC Oral Health. 2015 Nov 4;15:138. doi: 10.1186/s12903-015-0119-2 (PMC4632367; doi:10.1186/s12903-015-0119-2)
Supplement: Additional file 2: — Table. Job-seeking Skills Self-efficacy (JSS) scale. (DOCX 58 kb) [file 12903_2015_119_MOESM2_ESM.docx]

**Appendix 2**

**Table: Job-seeking Skills Self-efficacy (JSS) scale**

| **Items** | **Measures to address one’s perceived level of confidence** |
| --- | --- |
|  | **Independence skills** |
| 1. | Requesting a job application form? |
| 2. | Completing a job application form? |
| 3. | Creating a resume? |
| 4. | Traveling to the interview? |
| 5. | Working on your own? |
| 6. | Your interview skills? |
|  | **Social skills** |
| 7. | Presenting you at an interview? |
| 8. | Speaking during an interview? |
| 9. | Meeting new people? |
| 10. | Contributing to a meeting or discussion? |
| 11. | Working with a team? |
| 12. | Career advancement? |
